# The Halogenated Metabolism of Brown Algae (Phaeophyta), Its Biological Importance and Its Environmental Significance

**DOI:** 10.3390/md8040988

**Published:** 2010-03-31

**Authors:** Stéphane La Barre, Philippe Potin, Catherine Leblanc, Ludovic Delage

**Affiliations:** 1 Université Pierre et Marie Curie-Paris 6, UMR 7139 Végétaux marins et Biomolécules, Station Biologique F-29682, Roscoff, France; E-Mails: potin@sb-roscoff.fr (P.P.); leblanc@sb-roscoff.fr (C.L.); delage@sb-roscoff.fr (L.D.); 2 CNRS, UMR 7139 Végétaux marins et Biomolécules, Station Biologique F-29682, Roscoff, France

**Keywords:** halogen speciation, brown algae, defense metabolites, haloperoxidases, biogeochemistry

## Abstract

Brown algae represent a major component of littoral and sublittoral zones in temperate and subtropical ecosystems. An essential adaptive feature of this independent eukaryotic lineage is the ability to couple oxidative reactions resulting from exposure to sunlight and air with the halogenations of various substrates, thereby addressing various biotic and abiotic stresses *i.e.*, defense against predators, tissue repair, holdfast adhesion, and protection against reactive species generated by oxidative processes. Whereas marine organisms mainly make use of bromine to increase the biological activity of secondary metabolites, some orders of brown algae such as Laminariales have also developed a striking capability to accumulate and to use iodine in physiological adaptations to stress. We review selected aspects of the halogenated metabolism of macrophytic brown algae in the light of the most recent results, which point toward novel functions for iodide accumulation in kelps and the importance of bromination in cell wall modifications and adhesion properties of brown algal propagules. The importance of halogen speciation processes ranges from microbiology to biogeochemistry, through enzymology, cellular biology and ecotoxicology.

## 1. Introduction

Most organisms seem to have the ability to produce organohalogens, and the reported chemodiversity of this class of compounds (3,800 in 2003) has jumped 20-fold in the last 30 years [1]. Marine halocarbon producers encompass an extensive phyletic range, and sessile life forms (algae and invertebrates) use a rich repertoire of diffusible and mucus-bound molecules, many of which are halogenated, for communication in the largest sense. Biogenic halogen chemistry is thought to have arisen in prokaryotes in response to the emergence of photosynthesis, the oxygen enrichment of the atmosphere by cyanobacteria in archean times (some three billion years ago), and in response to the cellular generation of toxic reactive oxygen species (ROS) originating from one (*i.e.*, O_2_^•−^ or HO_2_^•^) and two-electron reduction of O_2_ (*i.e.*, H_2_O_2_). The call for efficient reducing biochemistries involved chlorine, bromine and iodine available in seawater, and the development of corresponding enzymatic machineries with haloperoxidases. Oxidative H_2_O_2_ detoxication strategies, e.g., catalases, offer an alternative to ROS reduction, but there is no mention of their co-occurrence in the Cambrian precursors of extant cyanobacteria [2]. Therefore, it is likely that the halogen chemistry of marine algae reflects an episode of their polyphyletic evolutionary history. On one hand, the latter has involved a primary endosymbiosis (the engulfing of a prokaryotic cyanobacterium by a heterotrophic host cell), which lead to the emergence of the Plantae, the green lineage (green algae and land plants) and its sister group the red algae. On the other hand, chromophytic algae evolved by an additional process termed secondary endosymbiosis [3] which includes the endosymbiotic uptake of a eukaryotic alga, presumably an ancestor of modern red algae. Brown algae belong to the Stramenopile lineage, with marine representatives including the unicellular diatoms. While phylogenomic studies have recently validated the various lineages, the evolutionary relationships between the lineages remain partly unresolved [4]. Surprinsingly, less than 1% of the secondary metabolites from brown algae contain bromine or chlorine compared with 7% of green algal compounds and 90% of those reported for red algae [5]. Regarding halogen chemistry, iodination is more frequent and chlorination is less frequent in brown algae metabolites than in the red and green lineages.

In this review, we describe the specificity of the evolution of the halogen metabolism in brown algae, pointing towards the emergence of strict specificity for the oxidation of iodide in the most evolved orders of Phaeophyceae, such as the Laminariales, and, consequently, the emergence of novel physiological adaptations with their ecological and atmospheric significance.

## 2. Halogenated Metabolites of Brown Algae

More than 1,140 metabolites have been reported in the Phaeophyceae in 2007 [6], out of about 3,000 from the three macroalgal lineages and of a total of about 15,000 marine molecules [7]. However, in contrast to red algae, (i) the number of novel structures in brown algae decreases every year [8], (ii) surprisingly few brown algal metabolites are halogenated, yet as end products or as potential intermediates, they are involved in a wide range of primary and secondary functions, some of which are not completely established and (iii) no specific pharmacological use has been found for brown algal halogenated metabolites. We will consider the various classes of halogenated metabolites found in brown algae with considerations of their biosynthetic origin where applicable (Table 1).

### 2.1. Halogenated alkanes

**Figure 1:**
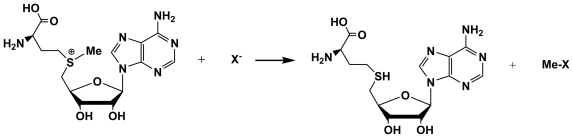
Methyltransferases mediate the transfer of a methyl group (Me) from SAM to a halide (X: halide ion). After [14].

**Figure 2:**
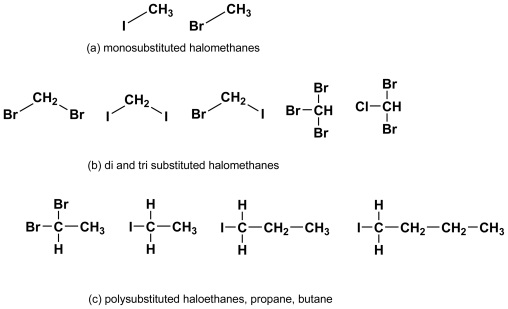
The most common halogenated alkanes found in effluxes from brown algae. (a) monosubstituted haloalkanes generated by action of SAM transferase; (b) and (c) polysubstituted alkanes supposedly generated as secondary products of V-BrPO action on enols. (see text).

Iodide (I^−^) and bromide (Br^−^) are actively taken up from seawater and (i) may directly undergo halogenation into monosubstituted halomethanes, or (ii) are oxidized respectively as HOI and HOBr by extracellular vanadium-dependent haloperoxidases (V-HPOs) which use photosynthetic hydrogen peroxide on a routine basis, and peroxide generated by oxidative burst under stress conditions, as discussed further. In the first instance, the biosynthesis of methyl bromide and methyl iodide involves the transferase - mediated nucleophilic attack of corresponding halides at the electrophilic CH_3_^+^S site of S-Adenosyl Methionine (SAM; Figure 1), but is clearly limited to monohalogenation [9,10], *i.e.*, the production of CH_3_I and CH_3_Br (Figure 2 (a). In the second case, methylation can produce di- and polysubstituted halomethanes, including a variety of hetero-substituted forms (e.g., CHBr_2_I; Figure 2 (b)) as in *Cystoseira barbata* [11]. Electrophilic reaction of I^+^ and especially Br^+^ with acceptors (centers of high electron density in e.g., enols, phenolics and phloroglucinol derivatives) can be rationalized on the basis of rearrangement of the double-bond. Hence a plausible mechanism is the successive bromination of the enols of C_2_ or C_3_ etc. units containing C=O groups followed by the loss of C_1_ (CH_2_O from CH_3_CHO and CH_3_CHO from CH_3_.CO.CH_3_ with concomitant formation of e.g., CH_2_Br_2_ and CHBr_3_ [10–12]. Whatever their origin (*i.e.*, by cationoid or anionoid halogenation), volatile halogenated organic compounds (VHOCs) globally predominate in brown kelp effluxes and ensure a fast disposal of the ROS detoxification products (HOI and HOBr) while conceivably regulating apoplastic iodine and bromine reserves (see below). Furthermore, excess HOI and HOBr resulting from vanadium-dependant bromoperoxidase (V-BrPO) upregulation under oxidative stress is thought to complement on site destruction of microbial infection by ROS, and prevent microbial biofilm formation [13] or modulate its architecture (La Barre, unpubl. results), while VHOCs would help discourage reinfection.

### 2.2. Tyrosine-bound halogens

**Figure 3:**
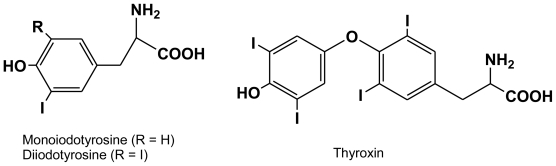
Mono- and diiodotyrosine are found in Laminariales, precursors to thyroxin (found as a thyroid hormone in some invertebrates and in higher animals).

Non-volatile iodine is mainly concentrated from seawater in the peripheral tissues of brown algae [15]. In Laminariales, several speciation studies have concluded that up to 90% of total iodine is mainly stored in a labile inorganic form identified as iodide [16–19], while its organic forms in *Laminaria* are dominated by hormone-like tyrosine derivatives, *i.e.*, monoiodotyrosine or MIT (10–15 ppm), diiodotyrosine or DIT (20–25 ppm), shown in Figure 3. The ubiquity of iodinated tyrosines (e.g., MIT, DIT) across extant eukaryotic phyla has shown their general function as endocrine molecules involved in cell-cell communication (plants) as well as time-coordinated and dose-dependent developmental changes, as reviewed by [20]. Trophic transfers of thyroxin (TH) and thyroid hormone precursors from primary producers (possibly detected in phytoplankton upon immunological assays) to consumers are essential to the metamorphosis of larvae of marine invertebrates [21] and gene regulation and signal transcription in vertebrates. Contrary to most organohalogenates of phaeophyte origin, tyrosin halogenations into MIT and DIT is believed to occur spontaneously, *i.e.*, they are not enzyme mediated [22]. The facile halogenation of MIT and DIT and the emphasis on the signaling role of these ubiquitous metabolites in eukaryotic physiology [20] makes them possible candidates as hormone-like substances, along with known elicitors (alginate hydrolysates) of kelps [23].

### 2.3. Halogenated phenolics

Phloroglucinol and derived products are essentially plant products. Singh and Bharate [24] conveniently separates them into phloroglucinols (mono-, di-, tri-, tetra- and oligomeric) and phlorotannins. According to this classification, only a few phloroglucinols have been isolated from macrophytic algae, whereas phlorotannins are exclusively found in brown algae and are regarded as the functional equivalents of terrestrial tannins. Halogenated monomeric phenolics are also occasionally found in brown algae, as well as in a few red algae.

#### 2.3.1. Halogenated phloroglucinols and phenols

**Figure 4:**
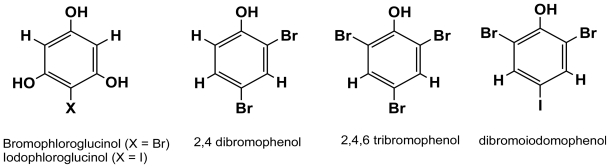
Halogenated phenolic monomers found in brown algae.

Iodophloroglucinol and bromophloroglucinol have been identified in the Laminariale *Eisenia arborea* [25]. Interestingly, Shibata *et al.* [26] have found that the Laminariales *Eisenia bicyclis* and *Ecklonia kurome* release monomeric bromophenols (2,4-dibromophenol, 2,4,6-tribromophenol and dibromo-iodophenol) in the surrounding medium (Figure 4), and retains oligomers and polymers in their tissues. Bromophenols are found in the peripheral tissues of a number of Australian brown algae [27] and are known to be much more efficient deterrents against grazing predators than phlorotannins, yet no study to date has correlated predation pressure with production and release of monomeric bromophenols in the water column.

#### 2.3.2. Halogenated phlorotannins

**Figure 5:**
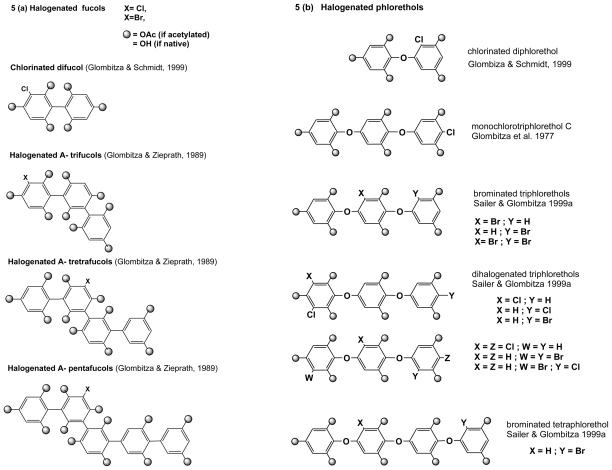

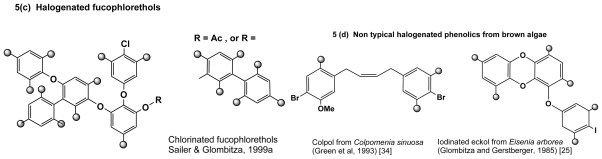
Halogenated phenolics from brown algae. (a)-Halogenated fucols found in the Laminariale *Analipus japonicus* (after [33]) and in the Sargassacea *Carpophyllum angustifolium* (after [32]). Spheres represent acetylation performed for spectroscopic analysis, or hydroxylation for native molecule. (b)-Halogenated phlorethols found in the Sargassacea *Cystophora reflexa* (after [29] (see also [32], and in *Laminaria ochroleuca* (after [31]). (c)-Halogenated fucophlorethols found in the Sargassacea *Cystophora reflexa* (after [29]). (d)-Non-typical halogenated phenolics from brown algae (after [34] and [25]).

According to linkage type, phlorotannins can be classified into four subclasses, *i.e.*, phlorotannins with an ether linkage (fuhalols and phlorethols), with a phenyl linkage (fucols), with an ether and a phenyl linkage (fucophlorethols), and with a dibenzodioxin linkage (eckols and carmalols), most of which have halogenated representatives in brown algae. The Fucales have been so far the best source of halogenated phlorotannins. For example, Koch and Gregson [28] have identified bromotriphlorethol in *Cystophora congesta*, and later Sailer and Glombitza [29] have isolated and characterized no less than 17 halogenated (brominated, chlorinated and iodinated) phlorotannins along with 30 non-halogenated forms [30] from the Sargassacea *Cystophora retroflexa*. One of these compounds, monochlorotriphlorethol was also found in the Laminariale *Laminaria ochroleuca* by Glombitza *et al.* [31]. The Sargassacea *Carpophyllum angustifolium*, in addition to chlorophloroglucinol, contains iododiphlorethol, chlorobifuhalol and chlorodifucol [32]. Monosubstituted eckols have been found in the Laminariale *Eisenia arborea*, along with halogenated phloroglucinols [25]. Ectocarpales have been little investigated, but chlorinated biaryl phloroglucinol derivatives (fucols) were found in *Analipus japonicus* [33]. Colpol, a non-typical phenolic derivative was isolated by Green *et al.* [34] in the Red Sea from a cosmopolitan Scytosiphonale, *Colpomenia sinuosa*. This brominated compound has a biphenyl butene structure, with strong cytotoxic activities. These various structures are shown in Figure 5 (a), (b), (c) and (d).

#### 2.3.3. Biosynthesis of halogenated phlorotannins

The biosynthesis of phenolics such as phloroglucinol with hydroxylations at the *meta* position are derived from the acetate-malonate pathway [35], in a process which may involve a polyketide synthase-type enzyme complex [36]. Chemically, condensation initiates intermolecular ring closure to yield hexacyclic ring systems (unstable triketide) which undergo tautomerization into the more stable aromatic form, phloroglucinol. Yet the exact biosynthesis pathway of phlorotannins is not known [37]. The involvement of apoplastic vanadium-dependent haloperoxidases, in the presence of halide ions and hydrogen peroxide has been demonstrated for *in vitro* cross-linking of phlorotannins [38,39], but the presence of halogenated species during the oxidation process is still speculative and is awaiting confirmation by kinetic studies and mass spectral analysis. Sulfated low molecular weight polyphenols are found in a wide range of brown algae [40], but mixed (sulfated and halogenated) substitutions have never been reported so far.

### 2.4. Halogenated fatty acids

**Figure 6:**
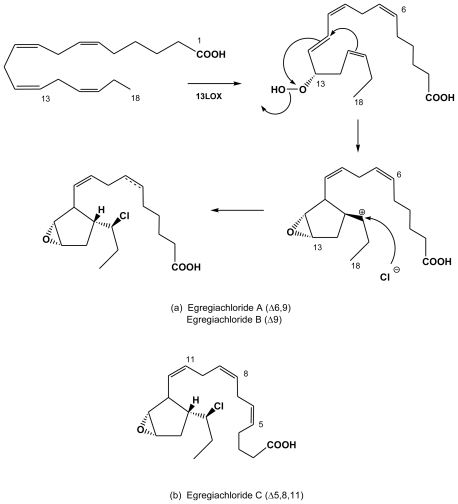
Halogenated eicosanoids (C18 oxylipins derived from stearidonic acid), isolated from the Laminariale *Egregia menziesii* [41]. (a) Egregiachloride A and B from stearidonic acic catalysis by 13LOX, cyclopentyl cyclization and subsequent carbocation attack at C16 by chlorine anion. (b) Egregiachloride C, similarly synthesized from eicosapentaenoic acid.

In brown algae, the overall composition in saturated and unsaturated fatty acids is equivalent to that found in red algae, with a high proportion of C18 and C20 polyunsaturated forms (PUFA). Three chlorinated oxylipins were isolated from the Laminariale *Egregia menziesii* (Alariaceae) by Todd *et al.* [41], two originating from the C18 PUFA stearidonic acid (egregiachloride A and B, Figure 6a) and one from the C20 PUFA eicosapentaenoic acid (egregiachloride C, Figure 6b).

**Figure 7:**
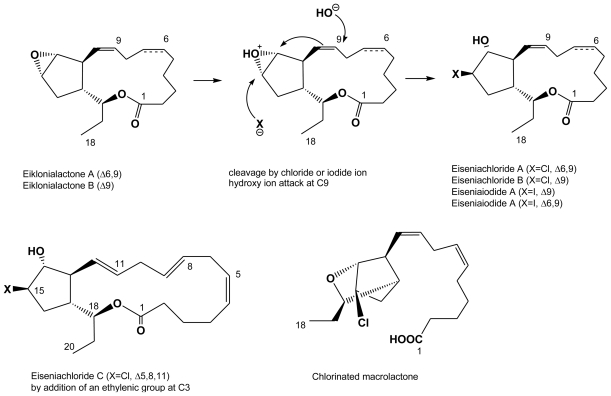
Halogenated eicosanoids (C18 oxylipins derived from stearidonic acid), isolated from the Laminariale *Eisenia bicyclis* [42].

Kousaka *et al.* [42] isolated six halogenated (eiseniachlorides) or iodinated (eiseniaiodides) species among a whole series of C18 oxylipins from another Alariaceae, *Eisenia bicyclis*, also biosynthesized from a stearidonic acid precursor. Along with eiseniachloride A and B and eiseniaiodide A and B, two cyclization variants (Figure 7), were determined by extensive 2-D NMR analysis. Interestingly, the halogenation (*via* C13 HPOTE resulting from LOX *i.e.*, lipoxygenase oxidation) occurs inside the cell and does not include brominated forms. Finally, a C20 form (*via* addition of an ethylene group) was determined as eiseniachloride C. Anti bacterial assays on *Bacillus subtilis* and on *Staphylococcus aureus* did not shown any improvement of the activities of any of the halogenated forms over the non-halogenated ones [42]. Ecklonialactones A and B are potential feeding repellents against predating abalone seashell, but no mention is made of this ecological role for their halogenated forms [42].

Similar to plants, in the brown alga *Saccharina (formerly Laminaria) angustata*, the LOX-derived fatty acid hydroperoxides can be further cleaved to form C-6 (derived from C18 PUFA) and C-9 aldehydes (derived from C20 PUFA) none of which have been reported under halogenated forms (see review [43]). Although chlorosulpholipids have been reported in a number of freshwater algae, there is no evidence that they occur in marine algae [44].

### 2.5. Halogenated terpenes

The only phaeophycean halogenated nor-sesquiterpenes were isolated from *Padina tetrastromatica* [45], possibly oxidation forms of perhydrolinalool. An atypical iodinated meroterpene (terpene with an aromatic moiety) has been characterized in *Ascophyllum nodosum* by Arizumi *et al.* [46]. This is in sharp contrast to red algae, in which secondary metabolites structures are abundant (over 1,200 structures in 2005), with highly diverse halogenated terpenes (over 800 chlorinated and/or brominated forms many of which have attracted the interest of pharmacologists [47]), and which probably reflect a capacity to diversify their defense strategies which is not encountered in brown algae.

### 2.6. Halogenated polysaccharides

If sulfatation of carbohydrates is a common feature in brown algae that confers them with specific physical and eco-physiological properties [48], there has never been any report of halogenated polysaccharides, in contrast to polyphenols in which either substitution (sulfates or halogen) can be found.

## 3. The Enzymology of Halogenation in Brown Algae

Nature’s strategies for incorporating halogens into organic molecules are multiple [49] and the discovery of new enzymes for halogenation has increased tremendously during the last decade [14] revealing new metalloenzymes, flavoenzymes, *S*-adenosyl-l-methionine (SAM)-dependent enzymes and others that catalyse halide oxidation using dioxygen, hydrogen peroxide and hydroperoxides, or that promote nucleophilic halide addition reactions [50]. In brown algae, it is now widely accepted that halogenation involves vanadium-dependent haloperoxidases (V-HPOs). Together with the heme-dependent haloperoxidases, V-HPOs utilize hydrogen peroxide to convert a halide ion (X^−^) to hypohalite (OX^−^) intermediate that is chemically equivalent to an electrophilic “X+”. They are named according to the most electronegative halide that they can oxidize, *i.e.*, chloroperoxidases (V-CPOs) can catalyze the oxidation of chloride as well as of bromide and iodide, bromoperoxidases (V-BrPOs) react with bromide and iodide, whereas iodoperoxidases (V-IPOs) are specific of iodide.

Vanadium-dependent haloperoxidases from marine sources are reviewed in this special issue [51], and a recent mini-review by Winter and Moore [52] provides an interesting phylogenetic tree of the different classes and sources of chloro- and bromoperoxidases found in nature. The first V-HPO was discovered in the fucoid brown alga *Ascophyllum nodosum* [53]. This enzyme has been extensively characterized as a bromoperoxidase at a biochemical and structural level [54,55]. As of today [14] and despite extensive researches on different V-BrPOs and V-ClPOs, the complete catalytic cycle is still uncertain and the origin of halide selectivity remains one of yet unanswered question [50,56]. Up to now, V-BrPO activities have been detected in both red and brown macroalgae, whereas V-ClPO are restricted to terrestrial fungi and to bacteria [52]. In microalgae, halogenation of organic compounds is known to involve halide methyl transferases [57] and no V-HPO has been yet identified on genomic data obtained from diatoms [58]. From algal genomic resources, several putative V-HPO homologues and dehalogenase enzymes have been identified in the red alga *Chondrus crispus* [59]. The first known V-IPO was identified and cloned in *Laminaria digitata* by [60] and phylogenetic analyses have shown that all brown algal V-HPOs present a monophyletic origin with a common ancestor with V-HPOs from red algae. However, it seems that in *L. digitata* the independent evolution of the two haloperoxidase gene families led to a novel biochemical function specialized in iodine oxidation [60]. Both V-HPO gene families seem to operate in a coordinate manner. Specific *vIPO* genes (coding for V-IPO enzymes) are upregulated following an oxidative burst to rapidly restore iodine loss, while some V-BrPO coding genes are specifically activated and likely to be involved in oxygen detoxification [61]. Recent proteomic studies on *Saccharina* (formerly *Laminaria*) *japonica* have revealed that V-BrPO are strongly upregulated during the summer months, in response to elimination of active oxygen species and protection of algal tissues from oxygen injury, as well as discouraging epiphytism which may seasonally hamper proper growth [62].

**Figure 9:**
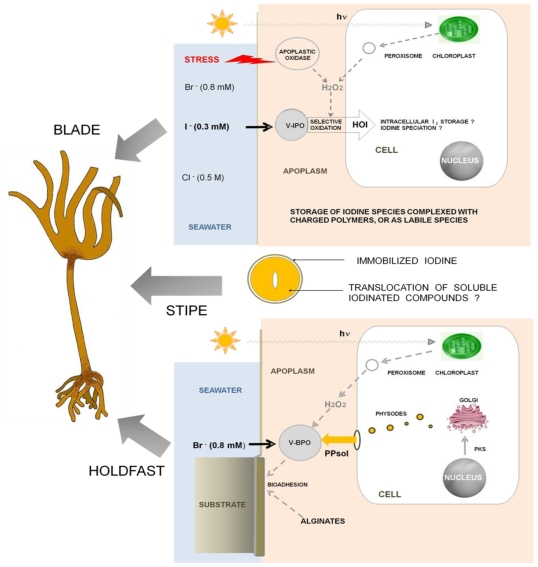
Halogen chemistry in *Laminaria digitata*. The different roles of V-HPO enzymes in brown kelp biology are conveniently contrasted in this diagram of a young whole thallus. In growing tissues of the blade (top), active uptake of iodine prevails as V-IPOs are upregulated to replenish iodine stocks to quench excess hydrogen peroxide (among other functions; see text). Iodine imaging revealed extracellular stocks associated with charged polymers for rapid bioavailability, rather than only intracellular I_2_stocks *via* HOI trans-membrane intake as hitherto believed. In the stipe (middle), mechanical resilience is maintained by outer cuticle hardening coinciding with the highest concentration of immobilized iodine. Metabolite translocation occurs in the softer inner matrix as well as signal molecules of suspected systemy. In the holdfast (bottom), soluble phlorotannins (PP) generated in the Golgi apparatus are contained in cytoplasmic physodes, which burst out in the apoplastic space. Bioadhesion is mediated by V-BrPOs, which cross-link the released PP in the presence of hydrogen peroxide and halide ions. The adhesive is thought to result from macromolecular scaffolds between the oxidized polymers and cell wall alginates.

In brown algae, V-HPOs and halides are likely to play a central role in oxidative detoxication, cell wall strengthening, and chemical defense in a context of cellular defense, as well as in substrate adhesion in young sporophytes. These various functions of V-HPOs crystallize large basic and applied interests, (see reviews in [50,51]). In Figure 9, the various biological functions of V-HPO are highlighted and contrasted in the various thallus parts in which they prevail, *i.e.*, bioadhesion at the holdfast region, defense against epiphytism, predation and biofouling being optimally expressed at the peri-meristematic region of the blade.

## 4. The Function of Bromination in Brown Algal Adhesion

A model of oxidative cross-linking of secreted phenolics mediated *via* the catalysis of a vanadium bromoperoxidase was proposed about twenty years ago, based on some indirect evidences such as V-BrPO immunolocalization in adherent eggs and spores of fucoid brown algae [63,64]. Indeed, this poorly grounded hypothesis [65] was challenged later when sophisticated techniques were used to investigate cross-linking processes in brown algae. The cross-linking of brown algal phlorotannins catalyzed by the V-BrPO from *A. nodosum* was measured using the quartz crystal microbalance with dissipation monitoring (QCM-D) method [38]. Phlorotannins adsorbed to a quartz crystal sensor were shown to change their mechanical properties upon the addition of V-BrPO, KBr and H_2_O_2_. The decreased dissipation upon addition of the cross-linking agents was interpreted as intramolecular cross-links formed between different phloroglucinol units in the phlorotannins [38]. Additionnal measurements using surface plasmon resonance (SPR) and UV/Vis spectroscopy verified the results achieved with QCM-D that all components *i.e.*, V-BrPO, KBr and H_2_O_2_ were necessary in order to achieve intramolecular *in vitro* oxidative cross-linking of the polymers. Based on the occurrence of halogenated phlorotannins in brown algae, while in low abundance [66], the involvement of a V-BrPO in the catalysis of phenolic cross-linking is our favourite hypothesis. Hardening with alginate and/or calcium is essential for high cohesive strength as shown in shear-lap tests that measured the adhesion strength of this assemblage [67]. All formulations were capable of adhering to a variety of surfaces, however the adhesion properties were influenced by the halide used [39]. QCM-D results showed that the kinetics of the oxidation was faster with iodide than with bromide. Small angle X-ray scattering (SAXS), light scattering and cryo-Transmission Electron Microscopy (TEM) experiments [67] revealed that oxidized polyphenols self-assemble into chain-like objects, irrespective of the oxidation conditions [39]. Yet, slight differences in the aggregate size were detected. Moreover, oxidation with iodide generates stiffer networks, suggesting that the interaction between the alginate and the polyphenol could be the cause of the reduced adhesion [67]. When coupled with results showing that V-BrPO brings about curing of adhesive extracts more than other catalysts, these data implicate V-BrPO to be the key reagent in controlling the cross-linking of phenolic polymers for the assembly of brown algal adhesives. These conclusions were confirmed by independent studies investigating the interaction between polyphenolics from the brown alga *Padina gymnospora* and alginate from *Fucus vesiculosus* using size exclusion chromatography (SEC) and optical tweezers microscopy [68]. It revealed the formation of a high-molecular-weight complex only when V-BrPO was added, indicating that the enzyme is essential for the binding process, along with a decrease in alginate viscosity. However, the covalent binding of uronic acid residues with some aromatic rings suggested by Salgado *et al.* [68] are not yet demonstrated in brown algae and SAXS and cryo-TEM measurements disfavoured this hypothesis (for a detailed discussion see Potin & Leblanc [69]). In this context, oxidized bromine or iodine in brown algae participates in a number of reactions with organic substrates, in which the resulting metabolites are involved in primary (oxygen detoxication, tissue repair, bioadhesion) and secondary (surface antibacterials) roles.

## 5. The Role of Iodine in Brown Algae

### 5.1. The emergence of specificity for iodine oxidation in brown algae

The element iodine has been discovered in the ashes of brown algal kelps in the beginning of the 19th century. The kelp *Laminaria digitata* presents an average iodine content of 1.0% of dry weight, representing a ca. 30,000-fold accumulation of this element from seawater [70]. In this brown alga, micro-chemical imaging of iodine distribution reveals an unexpected sub-cellular distribution in the cell walls of peripheral cells [15], with the predominance of iodide species [71]. Although *L. digitata* represents one of the most studied models, the biochemical pathways leading to this very efficient iodide trapping and the mechanism responsible for its release into the surroundings are still speculative. Specific oxidation processes are essential in both the mechanisms of iodine uptake and release. In *L. digitata*, iodine is thought to be taken up by a mechanism based on the haloperoxidase-mediated oxidation of iodide, a mechanism which requires a constitutive apoplastic level of hydrogen peroxide of circa 50 μM [70] and which could involve an apoplastic V-IPO [60,72]. Recent studies [15,69] have shown that iodide is mainly chelated by apoplastic macromolecules. Such a storage mechanism should provide an abundant and accessible source of reduced iodine, which can be easily remobilized for potential anti-oxidative activities and chemical defense. This unique setup has never been described among all living systems. However, in the presence of exogenously-added 2 mM H_2_O_2_, a net efflux of iodide was observed [70]. Yet there is no clear answer as to why Laminariales have developed (or retained?) such an important and extensive metabolic dependence to iodine, as compared to most halogen-using marine life forms [71].

### 5.2. The function of the iodine metabolism

The molecular mass of iodine (126.90 U) is the highest by far of all elements used in biological systems, including metals. The prevalence of iodine chemistry in very early life forms has recently led to interesting speculations as its cornerstone role as a catalyst and inorganic antioxidant [20] without which the emergence of multicellularity would never have taken place. Indeed, iodine is regarded as an additional biomarker towards the presence of life in extraterrestrial environments. Early prokaryotes (LUCA) had to carry out the conversion of toxic environmental iodate into iodine which could be readily incorporated in the cytoplasm and perform a number of essential catalytic functions, e.g., by coupling with essential organic molecules. The reducing (electron loosing) capacity of halides being proportional to the atomic size of the halogen, the oxidation products of active oxygenated species (AOS) and iodide occur almost readily as compared to bromine or chlorine. Iodine production by iodoperoxidases in several phaeophyte taxa, e.g., several Laminariales, represent a very efficient way of quenching excess hydrogen peroxide and reactive oxygenated radicals, following oxidative burst, or simply accumulating in plastids from regular photosynthesis [71]. An equivalent functional (primary) role is assigned to highly nucleophilic phlorotannins which are produced in a number of shore Fucales, following seasonal patterns in western Brittany (France), especially during summer with longer exposures to sunlight, and at mid-tide levels [73]. Interestingly, it was shown that the average iodine contents were lowest in summer months (0.25–0.60% dry weight) and highest in late autumn and in winter (in the range 0.75–1.20% dry weight) in the kelp *Laminaria digitata* [74]. Taken together, these data may help understand how the two classes of compounds interplay as ROS quenchers. However, in *L. digitata*, soluble phlorotannins are very low [73] and therefore iodide is likely to play a major role as an antioxidant [71]. Further studies are required in other species of Laminariales and of Fucales in order to understand the respective roles and the possible alternation between phlorotannins and iodide. Within-thallus translocation has long been documented in red and various brown algae, the sieve elements in both lineages lacking the vascularized phloem structure of higher plants [75]. In Laminariales, translocation of carbon labeled photosynthates from older parts of the frond towards the meristematic region and to the holdfast haptera has been demonstrated [76]. Mannitol, malate, citrate, polar amino acids and proteins are non-selectively translocated *via* the sieve tube of *Macrocystis pyrifera*, as well as inorganic cations (K^+^, Na^+^, Mg^2+^ and Ca^2+^) and anions among which chloride and bromide [77]. Iodide was later found to translocate in a source-to-sink manner, compatible with known distribution of this element in Laminariales [78]. As of now, organic halides have not been identified as translocates, yet there are strong suspicions that regulation of V-HPO genes operates within the context of a systemic response [61]. Systemy would operate either externally (detection in the water column of an eliciting signal generated in a stressed region of the thallus by a naïve region of the same individual, followed by a defense response), or internally *via* translocation. It could involve halogenated compounds (VHOC) or oxygenated species (aldehydes) which are produced as defense compounds in plantlets of *L. digitata* which are metabolically challenged [79,80] and thus are possible pheromone-like candidates, along with known elicitors (alginate hydrolysates) of this particular kelp.

### 5.3. Environmental consequences of the iodine metabolism

**Figure 10:**
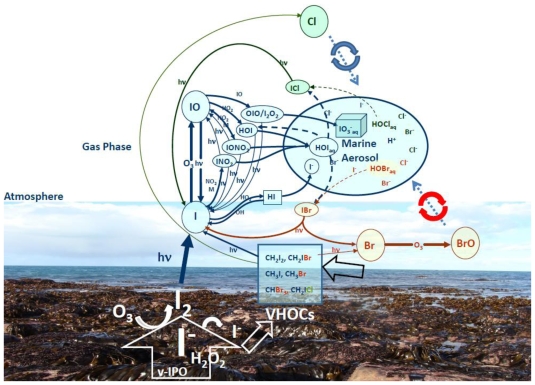
Iodine biogeochemistry at the Marine Boundary Layer. Schematic view of the link between kelp iodine species emissions and tropospheric iodine photooxidation, based upon current algal physiology, seaweed iodine speciation [71] and atmospheric chemistry [96] knowledge. The photograph features a kelp bed during a spring low tide in the vicinity of Roscoff. The large white arrow represents the contribution of kelps such as *Laminaria digitata* to the release of molecular iodine (I _2_) directly from its apoplastic and peripheral storage in the form of reduced iodide (I^−^). This oxidation is either catalyzed by endogenous specific vanadium iodoperoxidases (V-IPOs) using photosynthesis or stress- generated hydrogen peroxide (H_2_O_2_), or exogenously by the strong oxidant O_3_, when algae are exposed to air at low tide. These oxidative processes also lead to production of volatile halogenated organic compounds (VHOCs) by kelps (thin white arrow), although an estimated 300-fold lower than I_2_ fluxes [88]. Phytoplankton mainly contribute to I cycling through VHOC emission (black arrow above the sea). In the troposphere, atomic iodine (I) released by photolysis from I_2_ and photolabile VHOCs, reacts with available O_3_ to yield IO, rapidly establishing a photostationary state and consuming O_3_ whenever IO reacts either with itself (yielding OIO), HO_2_ (yielding HOI) or NO_2_ (yielding IONO_2_): rather than being re-photolysed to release I atoms. HOI and IONO_2_ may be taken up by aerosol particles, releasing the dihalogen species IBr, ICl or I_2_ via aqueous reaction with available Br^−^, Cl^−^ or I^−^, respectively, in the presence of sufficient acidity (H^+^). Iodine cycling is also linked to Br and Cl cycling, which speciate through similar chemical reactions in the gas phase leading to similar exchanges with marine aerosols.

Iodine species are present in the marine boundary layer (MBL) due to the release of I_2_ and of photo-labile iodocarbons from macro- and micro-algae (Figure 10). Whilst the primary source of inorganic chlorine and bromine species in the remote ocean is likely to be their release from the significant seasalt halide reservoir in atmospheric aerosol, the direct emission of halocarbons [81,82] from intertidal macroalgal species [79], from phytoplankton [83] and from biogenic marine aggregates [84] has been well established and contributes significantly to the coastal atmospheric burden of Reactive Halogen Species (RHS) [81,82]. Indeed shallow coastal areas where kelp beds of *Laminaria digitata* correspond to hotspots of biogenic halocarbon emission have been recently investigated with particular attention to iodine speciation. In particular, CH_2_I_2_ is the dominant organic iodine species released from kelps [81,82] and has it been suggested as one of the initiators of the iodine nanometric particles that function as cloud-forming nuclei [85]. The most widely-observed coastal inorganic RHS, iodine monoxide (IO), has also consistently been shown to exhibit a tidal signature, but only during daytime, consistent with photochemical formation of IO. In addition to the tidal halogen cycling, studies at Mace Head in Western Ireland revealed the rapid appearance of high concentrations of ultrafine aerosol particles at daytime low tide [86,87]. Should even a small fraction of the particles grow sufficiently large that they would act as Cloud Condensation Nuclei (CCN) at supersaturations corresponding to updraughts in typical marine stratocumulus clouds, such particle formation may significantly impact on local and regional radiative forcing and climate.

During the 2006 coastal campaign of the Reactive Halogens in the Marine Boundary Layer (RHaMBLe) project in Roscoff in Western France [88], it was clearly demonstrated that iodine-mediated coastal particle formation occurs, driven by daytime low tide emission of molecular iodine (I _2_), by macroalgal species fully or partially exposed by the receding waterline. Ultrafine particle concentrations strongly correlate with the rapidly recycled reactive iodine species, IO, produced at high concentrations following photolysis of I_2_. The heterogeneous macroalgal I_2_ sources lead to variable relative concentrations of iodine species observed by path-integrated [89] and *in situ* measurement techniques [90]. As shown at Roscoff, rockweed (the fucoid alga *A.nodosum*) beds may also emit more constantly than kelps because there are exposed to air more frequently (even during small tides) and for longer periods of time [91,92]. However, it was also observed at Cape Grim in Australia, that the contribution to particle formation of the fucoid alga *Durvillaea potatorum* is very limited because this alga does not release I_2_ [93]. These discrepancies have been tentatively explained by different capabilities of accumulation and emission between these two orders of large brown algae [92]. The evolution of new biochemical adaptations using more specific V-HPO has probably contributed to a better capacity to oxidize iodine and to use halides in kelps. In Laminariales (see section 2), iodoperoxidases activities that are specific for iodide, have been localized at least partly extracellularly in the apoplasm [60,94] and may explain the very high amount of iodide accumulated in their cell walls and higher efficiency of kelps for iodine oxidation, even in the absence of strong external oxidants, such as ozone. *Ascophyllum nodosum* was shown to possess two distinct isoforms of V-BrPO, one of them being located very superficially at the thallus surface [95] which is likely conferring a better capacity to oxidize iodide leading to high I_2_ emissions. Interestingly, this agree with the modeling of I_2_ emissions in the Roscoff area during the RHaMBLe project [92], which showed that the occurrence of rockweed in the mid-littoral zone of sheltered habitats complements efficiently the presence of large stands of Laminariales.

## 6. New Approaches, New Answers?

Marine halogenated natural products have important biological functions as signalling molecules and defense compounds for the producing organisms. If their therapeutic potential has attracted the attention of pharmacochemists worldwide since the early seventies [97], there is a now a strong interest in exploring the enzymes involved in their biosynthesis, in particular the vanadium-dependent haloperoxidases. Some V-HPOs have the ability to halogenate a range of organic compounds in a region- and stereospecific manner as evidenced in at least two orders of Rhodophyta [98] and by the wealth of bioactive chlorinated/brominated organic molecules found in this lineage [47]. In the Phaeophyta, the primary function of haloperoxidases is to prevent the accumulation of toxic oxygenated species (the vanadium-containing catalytic site uses two molecules of hydrogen peroxide).

### Ascophyllum nodosum

V-BrPOs still operate at 70 °C, in up to 60% organic solvents, they resist detergents, and maintain constant activity throughout the catalytic cycle (vanadium remains at VV oxidation state). This structural and functional resilience makes them all the more attractive as potential biocatalysts [52]. In addition, this particular enzyme is capable in vitro of carrying out sulfoxidation reactions enantioselectively on methyl phenyl substrate [99]. As biocatalysts of underwater “soft” or flexible adhesives, V-HPOs offer a very interesting alternative to “hard” or cementing biopolymers used by barnacles and many rock-dwelling molluscs. Bacterial haloperoxidases have been revealed by recent genomic studies. The chemistry of prokaryotic haloperoxidases may show new biological functions and thus greatly extend the potential of these enzymes as biocatalysts operating on novel and suitably selected organic substrates. In this area of research with potential impacts in the biosynthesis of marine natural products, it is very likely that structural biology and biotechnology will lead to important breakthroughs toward environment-friendly chemistry. To this end, methods such as exon shuffling for the directed evolution of bacterial and possibly algal enzymes will be employed.

## Figures and Tables

**Figure 8:**
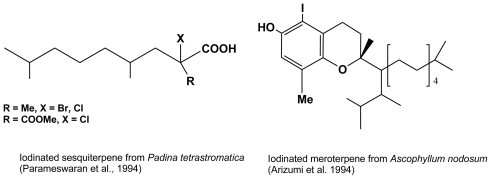
Halogenated linear nor-sesquiterpenes isolated from *Padina tetrastromatica* [45], and iodinated meroterpene isolated from *Ascophyllum nodosum* [46].

**Table 1:** Halogenated compounds from brown macrophytic algae reported in the literature. MIT and DIT = mono- and di-iodotyrosine; PG = phloroglucinol; PL = phlorethol; FC = fucol; FP= Fucophlorethol; FH = fuhalol; EK = eckol.

Species (Order)	Halogenated metabolite	Reference
**Laminariales (La)**	MIT, DIT	no specific reference
*Laminaria digitata*	volatile alkanes (Cl, Br, I)	Nightingale *et al.* (1995) [12]
*Laminaria ochroleuca*	monochlorotriPL C	Glombitza *et al.* (1977) [31]
*Laminaria ochroleuca*	monochlorotriphenol	Glombitza *et al.* (1977) (31]
*Egregia menziesii*	egregiachlorides A, B and C	Todd *et al.* (1993) [41]
*Eisenia bicyclis*	eiseniachlorides A, B and C	Kousaka *et al.* (2003) [42]
*Eisenia bicyclis*	eiseniaiodides A, B	Kousaka *et al.* (2003) [42]
*Eisenia arborea*	PG (I), EK (I)	Glombitza & Gerstberger (1985) [25]
**Fucales (Fu)**		
*Cystoseira barbata*	halogenated alkanes	Milkova *et al.* (1997) [11]
*Cystophora congesta*	bromotriPL A2	Koch & Gregson. (1984) (28]
*Cystophora reflexa*	mono, di, tri, tetraPL (Cl, Br, both), FP (Cl)	Sailer & Glombitza (1999)a [29]
*Ascophyllum nodosum*	iodinated meroterpene	Arizumi *et al.* (1994) (46]
*Carpophyllum angustifolium*	diFC (Cl), PG (Cl), diPL (I), biFH (Cl)	Glombitza & Schmidt (1999) [32]
**Dictyotales (Di)**		
*Padina tetrastromatica*	Cl and Br sesquiterpenes	Parameswaran *et al.* (1994) [45]
*Colpomenia sinuosa*	colpol (1,4 diphenylbutene 2)	Green *et al.* (1993) [34]
**Ectocarpales (Ec)**		
*Analipus japonicus*	tri, tetra and pentaFC	Glombitza & Zieprath (1989) [33]

Note: letters A, B, C refer to identified egregiachloride structures as shown in Figure 6 and to identified eiseniachloride and eiseniaiodide structures as shown in Figure 7.
